# Antioxidant Activities of Photoinduced Phycogenic Silver Nanoparticles and Their Potential Applications

**DOI:** 10.3390/antiox12061298

**Published:** 2023-06-18

**Authors:** Vijayakumar Maduraimuthu, Jayappriyan Kothilmozhian Ranishree, Raja Mohan Gopalakrishnan, Brabakaran Ayyadurai, Rathinam Raja, Klaus Heese

**Affiliations:** 1 Centre for Advanced Studies in Botany, University of Madras, Guindy Campus, Chennai 600 025, Tamil Nadu, India; vijayakumar.mk13@gmail.com (V.M.); jayappriyan@unom.ac.in (J.K.R.); rgrajamohan@yahoo.com (R.M.G.); algalpraba@gmail.com (B.A.); 2 Research and Development Wing, Bharath Institute of Higher Education and Research (BIHER), Sree Balaji Medical College and Hospital (SBMCH), Chennai 600044, Tamil Nadu, India; raja.research@bharathuniv.ac.in; 3 Graduate School of Biomedical Science and Engineering, Hanyang University, 222 Wangsimni-ro, Seongdong-gu, Seoul 133-791, Republic of Korea

**Keywords:** *Ulva lactuca*, light, silver nanoparticles, photocatalytic dye degradation

## Abstract

While various methods exist for synthesizing silver nanoparticles (AgNPs), green synthesis has emerged as a promising approach due to its affordability, sustainability, and suitability for biomedical purposes. However, green synthesis is time-consuming, necessitating the development of efficient and cost-effective techniques to minimize reaction time. Consequently, researchers have turned their attention to photo-driven processes. In this study, we present the photoinduced bioreduction of silver nitrate (AgNO_3_) to AgNPs using an aqueous extract of *Ulva lactuca*, an edible green seaweed. The phytochemicals found in the seaweed functioned as both reducing and capping agents, while light served as a catalyst for biosynthesis. We explored the effects of different light intensities and wavelengths, the initial pH of the reaction mixture, and the exposure time on the biosynthesis of AgNPs. Confirmation of AgNP formation was achieved through the observation of a surface plasmon resonance band at 428 nm using an ultraviolet-visible (UV-vis) spectrophotometer. Fourier transform infrared spectroscopy (FTIR) revealed the presence of algae-derived phytochemicals bound to the outer surface of the synthesized AgNPs. Additionally, high-resolution transmission electron microscopy (HRTEM) and atomic force microscopy (AFM) images demonstrated that the NPs possessed a nearly spherical shape, ranging in size from 5 nm to 40 nm. The crystalline nature of the NPs was confirmed by selected area electron diffraction (SAED) and X-ray diffraction (XRD), with Bragg’s diffraction pattern revealing peaks at 2θ = 38°, 44°, 64°, and 77°, corresponding to the planes of silver 111, 200, 220, and 311 in the face-centered cubic crystal lattice of metallic silver. Energy-dispersive X-ray spectroscopy (EDX) results exhibited a prominent peak at 3 keV, indicating an Ag elemental configuration. The highly negative zeta potential values provided further confirmation of the stability of AgNPs. Moreover, the reduction kinetics observed via UV-vis spectrophotometry demonstrated superior photocatalytic activity in the degradation of hazardous pollutant dyes, such as rhodamine B, methylene orange, Congo red, acridine orange, and Coomassie brilliant blue G-250. Consequently, our biosynthesized AgNPs hold great potential for various biomedical redox reaction applications.

## 1. Introduction

Research on nanomaterial synthesis is a fast-growing field due to the ever-expanding application prospects of nanomaterials. The production of various metal nanoparticles (NPs) has garnered attention due to their unique electronic, mechanical, optical, magnetic, and chemical properties, which differ from their respective bulk forms primarily due to an increased surface area-to-volume ratio [1,2,3]. Although several methods have been reported for nanoparticle synthesis, hydrochemical, sonochemical, chemical reduction, gamma-ray irradiation, micro-emulsion, laser ablation, electrochemical reduction, microwave irradiation, and photochemical reduction methods are the most commonly employed. However, all the above methods have several disadvantages, including high-energy consumption, high operational cost, and the release of hazardous wastes, leading to numerous environmental concerns [4]. Hence, interest in biological approaches, as opposed to physical and chemical methods, has increased due to their sustainability, biocompatibility, cost-effectiveness, and the fact that they do not require toxic chemicals or high energy. Furthermore, the regulation of particle size, shape, and stability of nanoparticles synthesized through biological processes is comparable to those synthesized using physical or chemical methods. Biological NPs synthesis involves extracts from bacteria, fungi, actinomycetes, algae, or plants [4]. Phytochemicals present in these biological entities act as both reducing and stabilizing agents, making biological agents ideal for metal nanoparticle synthesis at room temperature and with spontaneously controlled pH. Recently, “phyconanotechnology” has emerged as an exciting area of nanoscience that explores the synthesis and application of algae-mediated NPs. Both live and dead biomass of algae, referred to as “bionanofactories”, are being used for the synthesis of NPs [5]. Microalgae and macroalgae, as photoautotrophic organisms, have been extensively employed in the green synthesis of nanoparticles. Algae exist in various habitats and produce variegated bioactive substances such as polysaccharides, pigments, proteins, minerals, phenolics, flavonoids, tannins, and steroids [6], which are capable of reducing metal salts to respective NPs without any hazardous byproducts [7,8,9]. Recently, several studies have reported the synthesis of NPs using algal extracts from various algal species, including *Turbinaria conoides* [10], *Sargassum polycystum* [11], *Laminaria japonica* [12], *Botryococcus braunii* [5], *Oscillatoria limnetica* [13], *Chlorella ellipsoidea* [2], *Gracilaria edulis* [14], and *Synechocystis* sp. [15].

While the utilization of green synthesis for the production of NPs presents numerous advantages in the realm of biomedical applications, it is important to acknowledge that the procedure is typically characterized by its time-intensive nature [16]. Therefore, it is essential to find new methods or processes that can shorten the reaction time. The search for new or improved approaches has attracted the attention of researchers toward photo-mediated processes. Photo-mediated green techniques are becoming more popular because they are cost-effective and eco-friendly, provide control over the rate, size, and shape of silver NPs (AgNPs), and allow light to interact directly with biomolecules without requiring additional light absorption promoters [17,18]. Numerous articles have delved into the fascinating world of AgNP biosynthesis through a photo-mediated route. In one captivating study, Kumar et al. harnessed the power of direct sunlight to craft AgNPs, utilizing an aqueous extract of *Erigeron bonariensis* [17]. Meanwhile, Singh et al. astounded the scientific community by swiftly synthesizing AgNPs through a sunlight-induced pathway, employing an aqueous extract of *Dunaliella salina* [19]. Bhardwaj et al. explored the intricate realm of photochemical synthesis, employing an oyster mushroom extract and exposing it to the brilliance of direct sunlight [16]. Furthermore, Mankad et al. contributed to this exciting field with their extensive investigation into the sunlight-mediated green synthesis of AgNPs, utilizing *Azadirachta indica* leaf extract [20]. Lastly, Nguyen’s groundbreaking research shed light on the sunlight-driven synthesis of AgNPs, employing a pomelo peel extract [21].

Among various metal NPs, AgNPs are popular and important due to their facile purification and wide range of applications, including catalysis, biosensing, drug delivery, bioimaging, theranostics, food packaging, nonlinear optical devices, water treatment, antibacterial formulations, wound dressings, medicine, surgical instruments, and optoelectronics [18]. There is also considerable interest in employing AgNPs in degrading organic dyes. Dyes are synthetic organic substances widely used in the paper, plastic, leather, food, cosmetic, textile, and pharmaceutical industries. When effluents from industries that use dyes are discharged into water bodies without sufficient treatment, it leads to a decrease in dissolved oxygen levels and thereby threatens aquatic life [18]. Furthermore, untreated dyes released by industries contribute to eutrophication, the circulation of carcinogenic agents, and water-borne diseases [22]. Several methods exist for removing these non-biodegradable organic chemicals from the environment, including activated carbon sorption, flocculation, electro-coagulation, UV light degradation, and redox treatments [23]. However, the current situation necessitates better and improved wastewater treatment strategies due to the ineffectiveness of conventional and biological degrading approaches. AgNPs have been recently shown to be effective photocatalysts for degrading chemical complexes under ambient temperature and visible light irradiation. In addition to being inexpensive and rapid, this process can degrade whole contaminants into nontoxic and degradable byproducts [24]. The primary objective of this work is to understand the effect of light on the phycogenic biosynthesis of AgNPs and to assess the photocatalytic capacity of AgNPs in degrading hazardous pollutant dyes. We used the aqueous extract of *Ulva lactuca* as a reducing source for AgNP formation, where AgNP synthesis was mediated by both sunlight and normal LED white light. In addition, the biosynthesis of AgNPs was performed using a one-parameter-at-a-time approach to optimize the different variables involved in the synthesis. Parameters such as light intensity, wavelength, exposure time, and pH were taken into consideration in the synthesis of AgNPs. The experiment was also conducted under dark conditions to provide evidence of the crucial role of light in mediating AgNP biosynthesis.

## 2. Materials and Methods

### 2.1. Materials

All chemicals used in this research, including silver nitrate (AgNO_3_), were purchased from Sigma-Aldrich (St. Louis, MO, USA). Dyes such as Congo red (CR), methylene orange (MO), acridine orange (AO), rhodamine B (RB) and Coomassie brilliant blue G-250 (CBB G-250) were purchased from SRL chemicals (Mumbai, MH, India). All the reagents were at least 99% pure and used without further purification.

### 2.2. Collection of U. lactuca Seaweed

The green alga *U. lactuca* was collected from the Mandapam group of Islands in the Gulf of Mannar Marine Biosphere Reserve (southeast coast of Tamil Nadu, India; 8.49°–9.15° N, 78.11°–79.15° E). An authenticated voucher specimen, No. MUBL1039, was deposited in the Madras University Botany Laboratory Herbarium (MUBL), Center for Advanced Studies in Botany, University of Madras, Guindy Campus, Chennai, Tamil Nadu, India 600025. The collected samples were immediately brought to the laboratory in fresh plastic bags containing seawater to prevent dehydration. Algal materials were thoroughly washed with tap water, then finally with sterile double distilled water (ddH_2_O) to remove extraneous materials. They were then shade-dried for 5 days. They were subsequently oven-dried at 60 °C until a constant weight was achieved. The samples were then ground into a fine powder using an electric mixer grinder and stored at 4 °C until used.

### 2.3. Ulva lactuca Seaweed Extract Preparation

Seaweed aqueous extract was prepared by dissolving 10 g of *U. lactuca* powder in 100 mL of sterile ddH_2_O at 60 °C for 30 min under continuous stirring using magnetic pellets. The resulting mixture was then filtered using Whatman grade 1 filter paper (Sigma-Aldrich) and used immediately. A portion of the filtrate was also lyophilized and stored at 4 °C for further studies.

### 2.4. Phytochemical Content Analysis of U. lactuca Seaweed Extract

#### 2.4.1. Estimation of Total Carbohydrates in *U. lactuca*

To 1 mL of the sample (10 mg in 100 µL dimethyl sulfoxide (DMSO) + 900 µL of ddH_2_O), 1 mL of 5% phenol and 5 mL of concentrated sulfuric acid (H_2_SO_4_) were added, and this solution was thoroughly mixed by shaking. The solution was then incubated for 15 min in a boiling water bath. After cooling, the absorbance of the solution was measured at 490 nm using an ultraviolet-visible light (UV-vis) spectrophotometer (Jasco V-730, Anatek services Pvt. Ltd., Chennai, Tamil Nadu, India; range: 300–800 nm). The amount of total carbohydrate was calculated using a standard graph prepared from D-glucose, and the values are expressed as g/100 g *U. lactuca* dry weight [25].

#### 2.4.2. Estimation of Protein Content in *U. lactuca*

Sample (1 mL) (10 mg in 100 µL DMSO + 900 µL of ddH_2_O) and 5 mL of Bradford’s reagent were mixed thoroughly. The absorbance was measured at 595 nm using a UV-vis spectrophotometer against a blank. The amount of protein was calculated using a standard graph with different concentrations of bovine serum albumin (BSA) [26].

#### 2.4.3. Estimation of Lipid Content in *U. lactuca*

First, 0.5 mL of concentrated H_2_SO_4_ was added to 1 mL of the sample (10 mg in 100 µL DMSO + 900 µL of ddH_2_O) and mixed well. The tubes were then placed in a boiling water bath for 10 min and allowed to cool at room temperature. Next, 5 mL of a phosphovanillin reagent was added to 0.2 mL of the sample, mixed well, and incubated for 30 min at room temperature. After the incubation period, the developed color was measured at 520 nm. A standard graph was determined using cholesterol, and the values are expressed as g/100 g *U. lactuca* dry weight [27].

#### 2.4.4. Estimation of Total Phenolic Content in *U. lactuca*: The Folin–Ciocalteau Method

The total phenolic content in the extract was determined using Folin–Ciocalteu reagent (FCR). Briefly, 1 mL of the sample (10 mg in 100 µL DMSO + 900 µL of ddH_2_O) was mixed with 2.5 mL of FCR (diluted 1:10 *v*/*v*) followed by 2 mL of Na_2_CO_3_ (7.5% *w*/*v*). The tubes were vortexed and incubated for 90 min at room temperature. Absorbance was measured against a blank at 750 nm using a UV-vis spectrophotometer. The calibration curve was prepared using gallic acid equivalent (GAE) as a standard, and the total phenolic content of the extract was expressed as g GAE per 100 g of *U. lactuca* dry weight [28].

#### 2.4.5. Estimation of Flavonoids in *U. lactuca*: Aluminum Chloride Colorimetric Technique

The total flavonoid content of the extract was determined using 1 mL of the test sample (10 mg in 100 µL DMSO + 900 µL of ddH_2_O) and 4 mL of ddH_2_O in a volumetric flask (10 mL volume). After 5 min, 0.3 mL of 5% NaNO_3_ and 0.3 mL of 10% AlCl_3_∙6H_2_O were added. After 6 min incubation at room temperature, 2 mL of 1 M NaOH were added to the reaction mixture. The final volume was immediately adjusted to 10 mL with ddH_2_O. The absorbance was measured spectrophotometrically at 510 nm against the blank. A calibration curve was prepared using quercetin equivalents (g quercetin per 100 g of *U. lactuca* dry weight) [28].

### 2.5. Photocatalytic Biosynthesis and Optimization of AgNPs Using U. lactuca

Around 90 mL of 1 mM AgNO_3_ (16.96 mg in 100 mL ddH_2_O) was added to 3 separate conical flasks (250 mL). Then, 10 mL of seaweed extract solution was added to each flask. Each flask was kept under different conditions, including sunlight, normal white light (LED light) and complete darkness at room temperature for 24 h to observe the color change from a pale yellow to a brown solution, indicating the formation of AgNPs. The incubation time for the flask kept under dark conditions was extended to five days until AgNP formation was observed. Biosynthesis of AgNPs under different parameters, namely light intensity (5000, 10,000, 20,000, 30,000, and 40,000 lux), pH (5, 6, 7, 8, and 9), light wavelength (violet, red, green, and yellow) and exposure time (every 2 min, until 1 h), was evaluated. The synthesized AgNPs were subsequently subjected to centrifugation at 15,000 rpm for 20 min at 4 °C, utilizing a cooling centrifuge (REMI C-24 PLUS; Remi Elektrotechnik Limited Pvt. Ltd., Mumbai, India). Following centrifugation, the supernatant was discarded, and the pellet, which contained the AgNPs, was meticulously collected and dried in a vacuum desiccator. The resulting nanoparticle powder was subsequently transferred to sterile Eppendorf tubes and stored for subsequent characterization and dye degradation applications. Furthermore, 10 mL of a 9:1 ratio of AgNO_3_ and *U. lactuca* extract mixed in a test tube was kept under dark conditions and subsequently exposed to light through pinholes to corroborate the influence of light on the synthesis of AgNPs.

### 2.6. Characterization of Photocatalytically Biosynthesized AgNPs

The optimally biosynthesized AgNPs were characterized using various relevant techniques. The biosynthesis of AgNPs was confirmed by surface plasmon spectra using UV-vis spectrophotometry (at a wavelength range of 300–800 nm). The hydrodynamic diameter and potential charge of the AgNPs were determined using nanoparticle analyzer equipment (Nanoparticle Analyzer SZ-100, Horiba Scientific Pvt. Ltd., Chennai, Tamil Nadu, India). Briefly, the powdered AgNPs were diluted tenfold using distilled H_2_O, sonicated for 15 min, and then transferred into U-type tubes for measurement at 25 °C. Fourier transform infrared spectroscopy (FTIR) was employed to analyze the reducing agent responsible for nanoparticle synthesis (IS50 Smart ITX, ThermoFisher Scientific Pvt. Ltd., Mumbai, Maharashtra, India). The spectra were obtained in the range of 400 to 4000 cm^−1^. Transmission electron microscopy (TEM) was utilized to determine the size and shape of the AgNPs, while selected area electron diffraction (SAED) was performed to analyze the crystalline nature of the synthesized nanoparticles. Additionally, energy-dispersive X-ray spectroscopy (EDX) analysis was carried out to determine the elemental configuration of the AgNPs. The sample preparation for high-resolution transmission electron microscopy (HRTEM) involved suspending powdered AgNPs in 1 mL of ethanol and sonicating them for 15 min. For imaging purposes, 20 µL of the NP suspension was then dropped onto a carbon-coated copper grid and air-dried. The resulting sample was examined using TEM (JEM 2100Plus, JEOL Limited Corporation, Tokyo, Japan) operating at 120 kV. The crystallinity of AgNPs was assessed using X-ray diffraction (XRD-D8 Advance Bruker, Berlin, Germany). Dried powder of AgNPs was coated on an XRD grid to be analyzed over a range of 20° to 80° (2θ) using Cu Kα radiation (wavelength of 1.5406 Å) generated at 30 kV and 30 mA, with a scan speed of 4 deg/min. Atomic force microscopy (AFM) was performed to analyze the surface morphology of AgNPs (MFP-3D Classic, Oxford Instruments Asylum Research, Santa Barbara, CA, USA).

### 2.7. Catalytic Redox Activity of Photocatalytically Biosynthesized AgNPs

The catalytic reduction-oxidation (redox) activity of AgNPs was investigated for the degradation of hazardous organic dyes, including CR, MB, RB, AO, and CBB G-250, in an aqueous solution. The biosynthesized AgNPs (10 mg) were added to 50 mL of each dye solution (1 mg/100 mL) prepared separately. The suspension was stirred using magnetic pellets in the dark for 30 min to ensure the equilibrium of the working solution before irradiation. Subsequently, the suspension was exposed to sunlight for 3 h. At regular intervals of 30 min, 3 mL aliquots of the suspension were collected, filtered, and the absorbance spectrum was measured using a UV-vis spectrophotometer. Dye solutions without the addition of AgNPs served as a control reference for each dye.

## 3. Results and Discussion

### 3.1. Photocatalytically Induced Biosynthesis of AgNPs Using U. lactuca Aqueous Extract

**Figure 1:**
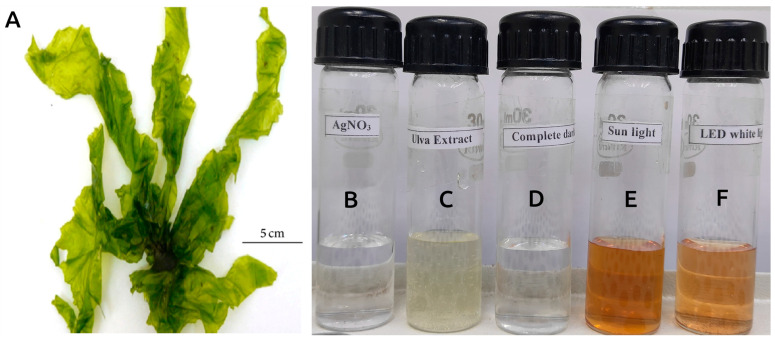
AgNPs were synthesized from the aqueous extract of *U. lactuca* via photoinduction (LU-AgNPs). (**A**) *U. lactuca* used for biosynthesis of AgNPs, (**B**) AgNO_3_ solution, (**C**) aqueous extract from *U. lactuca*, (**D**–**F**) *U. lactuca* extract treated with AgNO_3_ solution in the (**D**) dark, (**E**) sunlight, and (**F**) normal LED white light, respectively.

AgNPs were biosynthesized from the aqueous extract of *U. lactuca* via photoinduction (Figure 1). The *U. lactuca* extract was treated with an aqueous solution of AgNO_3_ (1 mM) in the presence of light, resulting in an immediate color change within a minute. The color of the Ag^+^/*U. lactuca* reaction mixture transitioned from pale yellow to brown as the reaction time increased, indicating a bio-redox reaction and formation of AgNPs. The synthesized materials are henceforth referred to as light-induced *U. lactuca*-mediated synthesized AgNPs (LU-AgNPs). The change in color of the reaction mixture is attributed to surface plasmon resonance (SPR), which involves the collective oscillation of electrons on the surface of metal particles [29]. This phenomenon leads to absorption spectra when the particles absorb light [30]. The SPR pattern is influenced by the size and shape of the metal particles, as well as the dielectric properties of the synthesis medium and the interactions between nanoparticles [31]. Moreover, the height of the SPR peak can be used to quantitatively determine the density of NPs in the solution [16]. AgNPs demonstrated a robust SPR peak in the visible spectrum, specifically in the wavelength range of 400 to 450 nm. The wavelength at which maximum absorbance occurred coincided with the plasmon peak, adding to the captivating optical properties of AgNPs.

**Figure 2:**
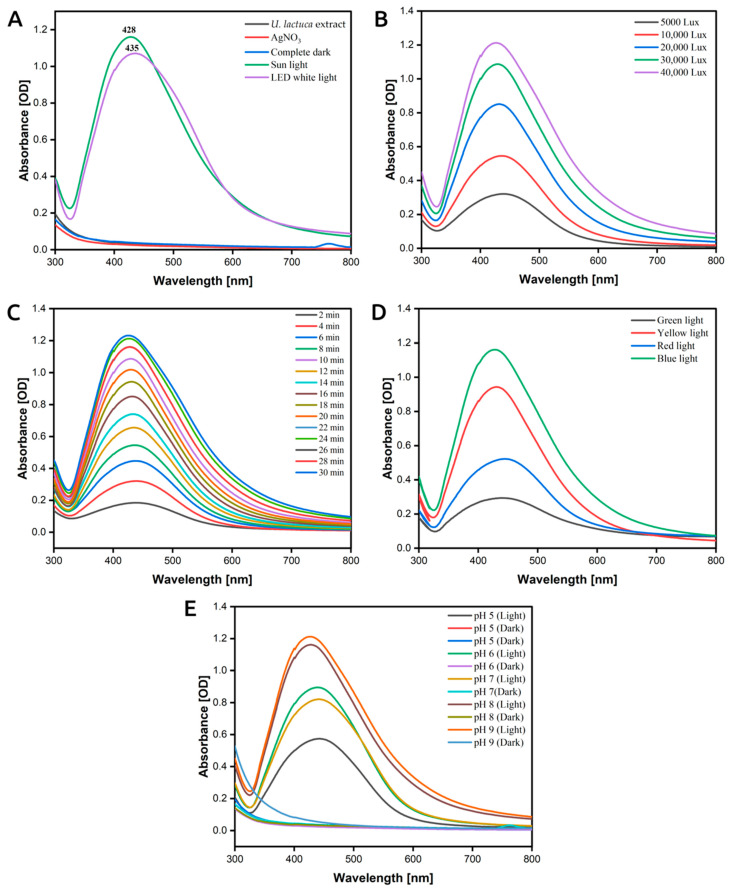
UV-vis spectra of biosynthesized LU-AgNPs. (**A**) LU-AgNP synthesis under sunlight, normal white light (LED), and under complete dark. AgNO_3_ solution (red) and aqueous extract from *U. lactuca* (black) served as references. (**B**–**E**) LU-AgNP synthesis under different sunlight intensities (**B**), at different time intervals under sunlight (**C**), at different wavelengths of sunlight (**D**), and at different pH values kept under sunlight and dark conditions (**E**).

Figure 2A depicts the UV−vis spectra of the LU-AgNPs prepared under sunlight and normal white light, showing characteristic SPR peaks at 428 nm and 435 nm, respectively. These results reveal that the LU-AgNPs prepared under normal white light are much more polydispersed compared to sunlight-mediated synthesis [18]. In comparison to earlier publications on the synthesis of AgNPs, Das et al. reported an absorbance peak at 447 nm for AgNPs synthesized using extracts of the outer shell fiber of *Cocos nucifera* under laboratory light conditions [32]. Neethu et al. confirmed the visible-light-induced synthesis of AgNPs by *Penicillium polonicum* through the SPR observed at 430 nm [33]. Singh et al. detected absorbance peaks at 440 and 431 nm for photoinduced biosynthesis of AgNPs from an aqueous extract of *Dunaliella salina* [19]. In another study, the synthesis of AgNPs by solar irradiation of cell-free *Bacillus amyloliquefaciens* extracts and AgNO_3_ exhibited an SPR peak at 423 nm [34]. These earlier reports indicate that sunlight-mediated synthesis shows a shorter SPR peak compared to normal white light-mediated synthesis. Contrastingly, the reaction mixture stored in a closed vessel in the dark did not show any SPR band, with no significant change in color for up to 5 days of incubation. Additionally, no absorption bands were observed either in the *U. lactuca* extract alone or in the AgNO_3_ solution. These results further emphasize the pivotal role of light in Ag^+^ reduction, in agreement with previous findings [17,32,35,36,37].

**Figure 3:**
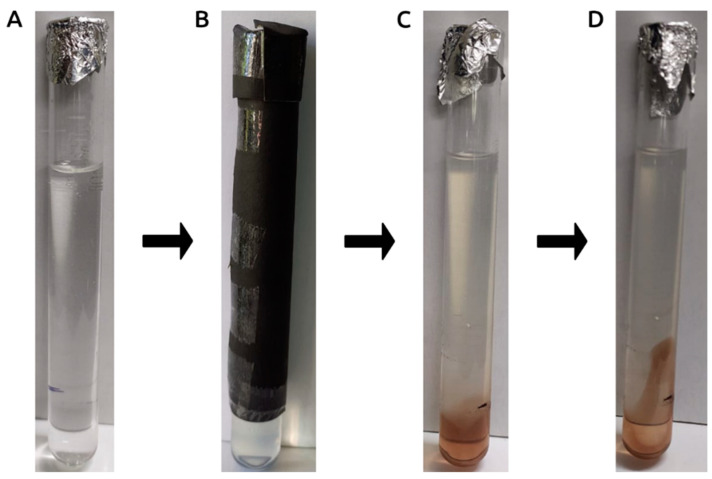
Simultaneous evaluation of photocatalytic LU-AgNP biosynthesis under opaque/light-exposed conditions. *U. lactuca* extract, along with AgNO_3_ (9:1), was simultaneously exposed to (**A**) 3/4 opaque conditions and (**B**) 1/4 light exposure. (**C**) LU-AgNP biosynthesis occurred only in the light-exposed portion of the test tube, and (**D**) dispersion of formed LU-AgNPs to the opaque portion occurred after manual disturbance of the test tube.

Remarkably, we shed light on the pivotal role of light induction in the biosynthesis of LU-AgNPs through a meticulously conducted experiment involving the passage of light through a test tube via a minuscule pinhole. This experiment unequivocally showcased that biosynthesis exclusively transpired within the realm exposed to radiant light, and gradually dissipated when subjected to disruption (Figure 3). Photo-mediated production of NPs is an efficient and environment-friendly process among various green synthesis methods [17,32,35]. Studies have previously highlighted the role of electromagnetic radiation from sunlight in facilitating the formation of green NPs [35,38]. For example, upon exposure to direct sunlight, the *Erigeron bonariensis* extract-AgNO_3_ solution changed color within a minute or less and later turned dark brown, indicating the formation of AgNPs [17]. These findings are consistent with the results of our current study. Hence, photo-assisted AgNP synthesis is a rapid, cost-effective, and productive method due to the short time required to complete the entire process. Recent work has demonstrated the rapid synthesis of AgNPs from red currant aqueous extract in the presence of sunlight irradiation [35]. Previous research has also provided insights into the possible mechanisms of sunlight-induced synthesis, the effect of sunlight intensities, wavelengths, and UV light-driven NP synthesis [35,39].

### 3.2. Phytochemical Composition of U. lactuca

Various phytochemical constituents in the aqueous extract of *U. lactuca* responsible for the reduction and capping of AgNPs were quantitatively analyzed. Table 1 presents the biochemical composition of *U. lactuca*, illustrating the influence of light on the biosynthesis of LU-AgNPs from *U. lactuca* extract. These phytochemicals are likely responsible for Ag^+^ reduction and act as capping agents, preventing aggregation and providing stability to the NPs [31]. Three characteristic electron-donating biocatalysts, namely proteins (including enzymes), polysaccharides and organic chemicals, could be involved in the reduction and capping of AgNPs [30]. The rapid synthesis of LU-AgNPs in the presence of light is attributed to the photosensitization of organic chemicals in the aqueous extract of *U. lactuca*, in which Ag^+^ cations utilize free electrons from phytochemicals such as proteins, saponins, quinines, polyphenols, flavonoids, and terpenoids [40] to reduce Ag^+^ cations to AgNPs [34]. Therefore, it is assumed that LU-AgNPs synthesized from the aqueous extract *U. lactuca* may be surrounded by these compounds, which could be beneficial for biomedical and pharmaceutical applications.

### 3.3. The Effect of Sunlight Intensity on AgNP Formation

In order to investigate the effect of different sunlight intensities on LU-AgNP biosynthesis, the reaction mixture was exposed to different light intensities for 1 h: 5000 lux, 10,000 lux, 20,000 lux, 30,000 lux, and 40,000 lux. As depicted in Figure 2B, the color of the reaction mixture changed from pale yellow to brown, with a darker hue observed at higher light intensities. The spectra of LU-AgNPs obtained under different light intensities showed λ max values ranging from 425 to 435 nm. The reduction rate of Ag^+^ ions to LU-AgNPs was relatively slow at lower intensities (5000 lux, 10,000 lux, and 20,000 lux), resulting in a lower SPR peak. This indicates slower photo-biochemical reaction kinetics for the nucleation and growth of LU-AgNPs. Additionally, a red-shift was observed in the absorption spectrum, suggesting larger-sized AgNPs at lower intensities. Conversely, at higher intensities (such as 30,000 lux and 40,000 lux), the reduction rate was faster, accompanied by a blue-shift in the absorption spectrum. The blue-shift in the SPR peak indicates that produced LU-AgNPs under high sunlight intensities have a smaller average size compared to those synthesized under low intensities. These observations indicate a direct correlation between light intensity and the bioproduction of AgNPs. Consequently, these experiments have paved the way for rapid photo-mediated biosynthesis of spherical-shaped AgNPs achieved at higher light intensities [19,41]. The reduction rate of Ag^+^ to Ag^0^ was significantly accelerated when exposed to higher light intensities. As a result, a sunlight intensity of 40,000 lux was selected for further experiments.

### 3.4. The Effect of Exposure Time on Photoinduced Biosynthesis of LU-AgNPs

NP stability and synthesis rate are time-dependent [42]. In the present study, UV-vis spectra were recorded every 2 min for 1 h under bright sunlight. It was observed that the absorbance increased steadily over time, reaching its maximum after 30 min of exposure (Figure 2C). The increased intensity of the SPR band indicates the continuous reduction of silver ions and the synthesis of isotropic AgNPs [41]. However, extending the exposure time beyond 35 min did not result in an increase in the band intensity, signifying the completion of the reaction. This was accompanied by a change in the physical appearance of the solution to a deep crimson brown. If an NP-containing solution retains its color over an extended period, it indicates that the NPs are evenly dispersed, and no further agglomeration has occurred [43]. The change in the solution’s color indicates a shift in surface resonance. SPR analysis was performed to determine the size, shape, and dispersion of photoinduced biosynthesized LU-AgNPs. A blue-shift in the SPR peak position corresponds to a decrease in particle size, while a red-shift indicates a larger particle size [44]. During photocatalytic LU-AgNP biosynthesis, a moderate blue-shift in SPR bands from 438 nm to 426 nm was observed up to 30 min, indicating a decrease in particle size with increasing reaction time. However, as the exposure time exceeded 30 min, the peaks became wider with a red-shift from 426 nm to 435 nm. This red-shift suggests a reduction in interparticle distances, indicating the biosynthesis of larger particles due to aggregation [41]. Therefore, 30 min of sunlight exposure was determined as the optimal time for LU-AgNPs biosynthesis and was used in further studies. Under the conditions used, control solutions of *U. lactuca* and AgNO_3_ did not exhibit the characteristic dark brown color and SPR band, indicating no abiotic reduction of AgNO_3_. These results align with the findings of Kumar et al., who synthesized AgNPs from an aqueous extract of *Erigeron bonariensis* [17].

### 3.5. The Effect of Wavelength on Photoinduced Biosynthesis of AgNPs

Besides light intensity, the wavelength is another critical factor for AgNP formation. Several investigations have been conducted to determine the effect of wavelength on the synthesis of AgNPs [16,45]. The presence of more photons of a particular wavelength in direct sunlight (the range of electromagnetic radiation emitted by the sun, extending from the ultraviolet to the infrared region) may have expedited the reducing activity and boosted the photocatalytic biosynthesis of AgNPs. Previous research demonstrated the sunlight-induced synthesis of AgNPs with citrus lemon extracts, suggesting that UV light in sunlight might be a possible source of light irradiation [46]. However, it has been mentioned that in many research, including ours, reaction mixtures contained in plastic tubes or glass vials do not transmit the UV portion of the sunlight [21,39,45]. Thus, blue light, which has less energy than UV light but more than other parts of the electromagnetic spectrum, may play a vital role in reducing Ag^+^ to Ag^0^ [47]. For a deeper understanding of the mechanisms and molecules responsible for the photoreduction of Ag^+^ to LU-AgNPs, spectral analysis of LU-AgNPs biosynthesized in color-wrapped reaction tubes was performed to study photon energy and the effect of wavelength of light on the photocatalytic biosynthesis of LU-AgNPs. Cellophane paper of various colors, including blue, red, green, and yellow, was used to cover the test tubes containing reaction mixtures that were exposed to sunlight for 30 min to check the efficacy of the different wavelengths of light in stimulating photocatalytic LU-AgNP biosynthesis. After exposure to different wavelengths of light, the reaction mixture changed from pale yellow to brown, indicating the reduction of Ag^+^ to Ag^0^ and the formation of LU-AgNPs. The SPR of the reaction mixture was determined using a UV-vis spectrophotometer with an absorbance range of 300 to 800 nm. Figure 2D shows the SPR spectra of LU-AgNPs biosynthesized by applying different wavelengths of light. The SPR band was observed between 428 and 446 nm in all cases. Different wavelengths of light caused variations in the maximum wavelength (λmax) and maximum absorbance. The SPR bands of all photocatalytically biosynthesized LU-AgNPs had a wavelength shorter than 446 nm. SPR band with a wavelength shift near 400 nm indicates small LU-AgNPs. The results revealed that the blue color-wrapped tube (380–450 nm) induced LU-AgNP biosynthesis with SPR spectra that correlated well with previous findings [16,48]. Interestingly, with a decrease in wavelength or an increase in light intensity, the SPR peak shifted toward a lower wavelength with an increase in SPR peak intensity. This study found that blue cellophane paper-wrapped test tubes exposed to sunlight produced smaller particles with a higher density. The current findings are congruent with those of Bhardwaj et al. [16]. Our study also revealed the highest absorbance, showing that this particular wavelength region of the visible spectrum promotes more LU-AgNP biosynthesis, followed by yellow, red, and green-wrapped tubes. It may be that the higher energy of blue waves of sunlight catalyzes the Ag^0^ reduction action by activating reducing moieties in the *U. lactuca* extract, leading to LU-AgNP biosynthesis. In accordance with the present study, Yang et al. [47] reported that blue light was the most effective among the red, green, blue, and yellow lights in the reduction of Ag^+^ ions. Moreover, Jayapriya et al. have described the possible mechanism for the photoreduction of Ag^+^ to Ag^0^, which involves the release of hydrogen atoms during the tautomerization of flavonoids under the incident of blue light in the extract, when the conversion of enol to the keto form may liberate reactive hydrogen atoms responsible for the reduction of Ag^+^ ions [45].

### 3.6. Effect of pH on Photoinduced Biosynthesis of AgNPs

The size and form of biologically produced NPs are substantially influenced by pH, since pH affects the electrical charge of biomolecules and capping agents, directly impacting their ability to bind and reduce metal ions [30]. The effects of different pH levels (i.e., 5, 6, 7, 8, and 9) on the photocatalytic biosynthesis of LU-AgNPs were studied under both light and dark conditions to determine the true cause of the reaction under both conditions. When exposed to light, all reaction mixtures with varying pH values exhibited the biosynthesis of LU-AgNPs, resulting in a color change from pale yellow to brown. However, the reaction mixture kept in the dark did not show any color change, indicating that pH adjustment alone cannot initiate the biosynthesis of LU-AgNPs in the absence of light. Figure 2E illustrates the UV-vis spectra of the reaction mixtures incubated at different pH conditions under light, showing characteristic absorption peaks between 425 and 450 nm. Highly acidic and highly basic pH conditions have been reported to hinder the reduction of Ag^+^ ions to AgNPs during the biosynthesis process [49]. However, as the pH increased from 5 to 9, the rate of LU-AgNP production increased but subsequently dropped when the pH exceeded 9 [50,51]. Based on spectral observations, the photocatalytic biosynthesis of LU-AgNPs was most efficient and rapid under alkaline reaction conditions (pH 8–9). Other researchers have also found that alkaline media provide a more favorable environment for AgNP production compared to acidic media, as the increase in negatively charged hydroxide ions (OH^–^) promotes the rapid reduction of Ag^+^ ions. In contrast, the slow rate of Ag^+^ ion reduction seen in the acidic medium might be due to electrostatic repulsion within the reaction mixture [52]. Furthermore, at acidic pH (5–6), there was a slight red-shift in the λmax (435 nm), indicating that the size of the produced LU-AgNPs tended to decrease as the pH value increased due to a rapid nucleation process that resulted in a large number of tiny particles. Other scientists made similar observations when examining the effect of pH on the biosynthesis of AgNPs (though without light) [53]. The effect of pH on the synthesis of LU-AgNPs can be attributed to the photo-oxidation of phenolic compounds, which releases H^+^ ions favoring the nucleation of AgNPs under alkaline conditions [54]. Moreover, at alkaline pH 9, smaller-sized NPs are formed, as confirmed by the blue-shift in the SPR band. Based on the obtained results, the optimal parameters for LU-AgNPs synthesis under direct sunlight were 40,000 lux intensity, 30 min exposure time, and pH 9. Therefore, these conditions were adopted for further LU-AgNPs synthesis, characterization, and dye degradation applications.

### 3.7. Characterization of Photoinduced Biosynthesis of AgNPs

After confirming the AgNPs through UV-vis spectrophotometry analysis, photocatalytic biosynthesized AgNPs were subjected to further characterization using TEM, SAED, EDX, AFM, FTIR, XRD, DLS, and zeta potential analyses.

#### 3.7.1. Transmission Electron Microscopy (TEM) of Photoinduced Biosynthesized AgNPs

**Figure 4:**
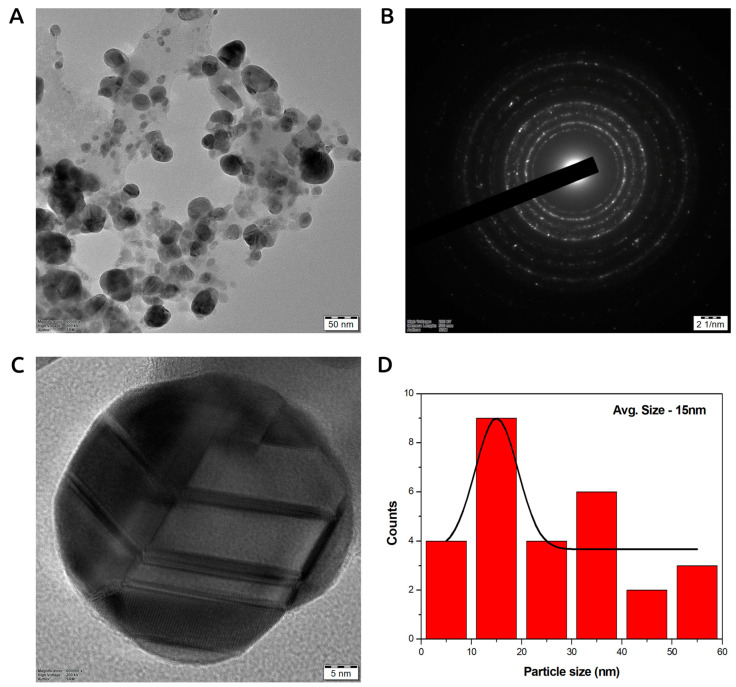
(**A**) Transmission electron microscopy (TEM) image of photocatalytic biosynthesized LU-AgNPs at 50 nm resolution, (**B**) SAED pattern of LU-AgNPs, (**C**) High-resolution transmission electron microscopy (HRTEM) image of a single spherical NP, and (**D**) histogram showing different size distributions of LU-AgNPs.

TEM was applied to characterize the size, shape, and morphology of photocatalytic biosynthesized LU-AgNPs. For TEM examination, a thin coating of ultrasonically dispersed material was applied on a copper grid and allowed to dry completely in a vacuum desiccator. Figure 4 shows TEM images of the produced AgNPs at various magnifications. The observed UV-vis spectral peaks and TEM pictures closely corresponded. The micrograph of photocatalytically biosynthesized LU-AgNPs revealed spherical particles that were well dispersed (Figure 4A). The selected area electron diffraction patterns of AgNPs exhibited concentric rings indexed to 111, 200, 220, and 311 planes of the face-centered cubic silver phase (Figure 4B). This suggests that the diverse wavelengths present in direct sunlight resulted in the synthesis of particles with a broad size distribution. Based on the obtained results, it is hypothesized that a single wavelength, specifically blue light, applied to the reaction mixture may possibly facilitate the synthesis of AgNPs with a narrow particle size range and with a high degree of mono-dispersity. This finding is consistent with a previous study by Bhardwaj et al. [16], which also reported that direct sunlight-mediated synthesis leads to particles with a broad size distribution, while a blue light-assisted synthesis resulted in a narrower particle distribution. Various previous other methods have utilized hazardous chemicals to synthesize monodispersed AgNPs [55,56]. However, our optimized biogenic process can yield monodispersed AgNPs, offering a unique, rapid, cost-effective, eco-friendly, and environmentally benign alternative approach [16]. The presence of a prominent circular ring in the SAED pattern further indicates the crystalline structure of the spherical photocatalytically biosynthesized LU-AgNPs. The HRTEM image revealed lattice fringes with an interplanar separation of 0.23 nm, corresponding to the 111 planes of face-centered cubic AgNPs (Figure 4C). The size distribution histogram corresponding to the TEM image showed that the sizes of LU-AgNPs ranged between 5 and 40 nm, with an average size of 15 nm (Figure 4D). These achieved small sizes of AgNPs are comparable to those produced by powerful reducing agents like NaBH_4_ [57] and N_2_H_4_ [58]. The histogram reveals that the majority of LU-AgNPs are concentrated in a size range of 10 nm to 15 nm, showcasing their maximum distribution. A similar size range was reported by other researchers who used an aqueous extract of *Dunaliella salina* for the photocatalytic biosynthesis of AgNPs [19].

#### 3.7.2. Energy-Dispersive X-ray Spectroscopy (EDX) of Photoinduced Biosynthesized AgNPs

**Figure 5:**
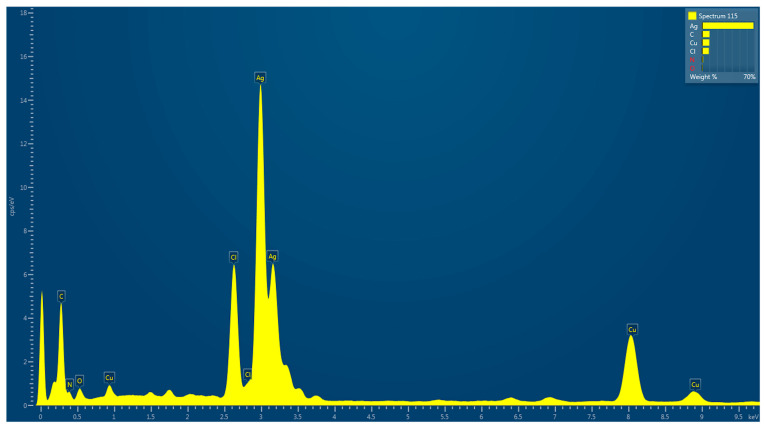
Energy-dispersive X-ray (EDX) spectrum of photocatalytic biosynthesized LU-AgNPs confirming nanoparticle synthesis.

Energy-dispersive X-ray analysis was used to determine the elemental composition of LU-AgNPs. The optical absorption peak detected at 3 keV confirmed the presence of AgNPs, which is typical for surface plasmon resonance absorption of metallic silver nanocrystals, as shown in Figure 5. A peak between 2.7 and 3.5 keV indicates the formation of AgNPs from SPR [59]. The greater proportion of Ag, as revealed by elemental analysis, served to validate that the composition of the AgNPs consists predominantly of silver. In addition to Ag, peaks corresponding to biomolecules such as chlorine (Cl), carbon (C), copper (Cu), traces of oxygen (O), and nitrogen (N) were detected. The presence of nitrogen and oxygen confirmed that the organic compounds from the extract were adsorbed on the surface of the metallic LU-AgNPs, as algal phytochemicals act as both reducing and capping agents for biosynthesized AgNPs [60]. The carbon and copper peaks were attributed to the carbon-coated copper grid used in the EDX analysis. Consistent with the findings of this study, a typical optical absorption peak associated with metallic Ag was observed at 3 keV in the EDX spectrum of AgNPs synthesized from *Chlorella ellipsoidea* [2]. In another study, the elemental composition of AgNPs synthesized from *U. lactuca* exhibited varying quantities of elements, such as carbon, chlorine, potassium, manganese, oxygen, and sodium [61]. These findings closely align with the present study. Similarly, Chien et al. reported the presence of carbon and oxygen in the EDX spectrum of green-synthesized NPs, which is similar to our study [62]. Therefore, the different elements observed in the LU-AgNPs spectrum may represent minerals present in the *U. lactuca* extract that surround the AgNPs during synthesis.

#### 3.7.3. Atomic Force Microscopy (AFM) of Photoinduced Biosynthesized AgNPs

**Figure 6:**
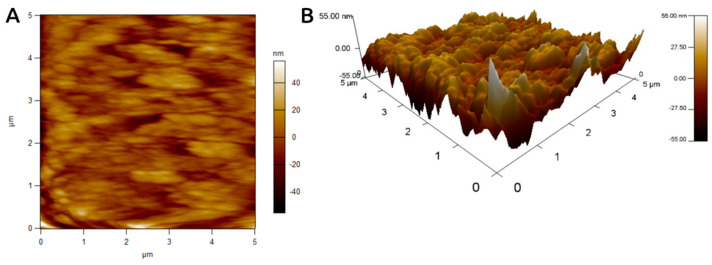
2D and 3D atomic force microscopy (AFM) images displaying topographical features (such as homogeneity, spherical shape, and dense packing) of photocatalytic biosynthesized LU-AgNPs.

The surface texture of photocatalytically biosynthesized LU-AgNPs was examined using AFM. Figure 6 displays the two-dimensional (2D) and three-dimensional (3D) topographical AFM images. Unlike electron microscopes, AFM provides 3D images of nanoparticles. It offers high resolution and does not require conductive samples or high-pressure vacuum conditions. A 3D AFM image revealed that the LU-AgNPs occur as homogeneous, polydispersed, spherical, and densely packed, like tiny grains across the entire scanned region’s surface. The AFM images further confirmed the broad size distribution of the synthesized LU-AgNPs due to the different wavelengths involved in the direct sunlight-mediated synthesis. These findings are consistent with our TEM images of LU-AgNPs. The 3D images of the NPs indicated a height of 55 nm. Consistent with the present study, Prathna et al. reported the green synthesis of stable AgNPs using citrus lemon extract induced by sunlight irradiation. Their AFM images showed spherical shapes of NPs with sizes <100 nm [46]. The white patches in the 3D image depict the surface roughness caused by certain organic compounds in the LU-AgNPs, which are also responsible for their reduction and capping.

#### 3.7.4. Fourier Transform Infrared Spectroscopy (FTIR) of Photoinduced Biosynthesized AgNPs

FTIR spectral analysis was carried out to determine the involvement of functional groups in the bioreduction of Ag^+^ ions and the stabilization of photocatalytically biosynthesized LU-AgNPs [63].

**Figure 7:**
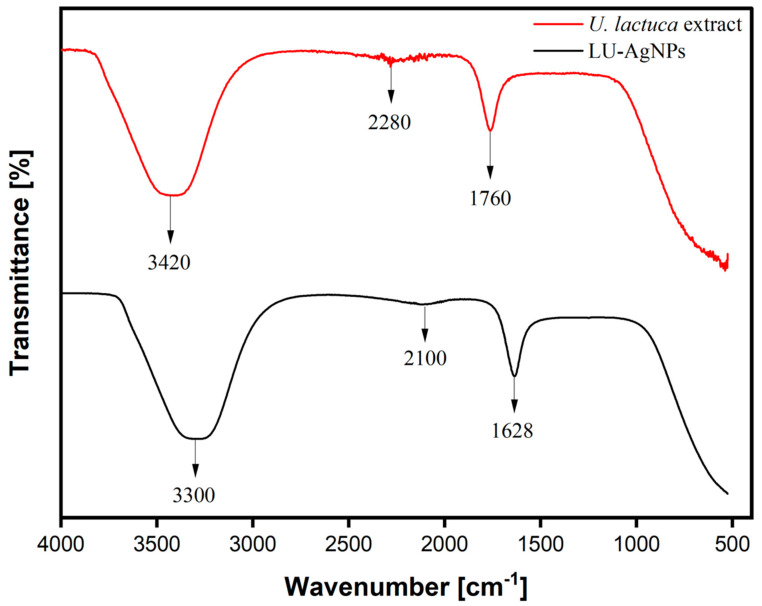
The Fourier transform infrared spectroscopy (FTIR) spectrum of *U. lactuca* extract shows major peaks representing hydroxyl, primary amine (O–H and N–H bonds, peak at 3420 cm^−1^), alkyne (C=C bonds, peak at 2280 cm^−1^), and vibrational stretching carbonyl (C≡C bonds, peak at 1760 cm^−1^) groups. FTIR spectrum of LU-AgNPs shows peaks that represent hydroxyl, primary amine (O–H and N–H bonds, peak at 3300 cm^−1^), terminal alkyne (C=C bonds, peak at 2100 cm^−1^), and primary amide (N–H stretching and C=O bending vibration at 1628 cm^−1^) groups.

The FTIR spectra of *U. lactuca* aqueous extract and LU-AgNPs displayed peaks at different wavelengths corresponding to biomolecules that contributed to capping and bioreduction during the synthesis of these LU-AgNPs. Hence, the FTIR spectrum of *U. lactuca* extract showed major peaks at 3420 cm^−1^, 2280 cm^−1^, and 1760 cm^−1^ (Figure 7). The broad, intense peak at 3420 cm^−1^ corresponds to the strong stretching vibration of hydroxyl groups (O–H) of the phenols and flavonoids [13], while the N–H stretching vibration represents the peptides and proteins and intramolecular H-bonds from water molecules [64]. A minor peak was observed at 2280 cm^−1^, attributed to stretching vibrations of alkynes (C≡C), likely derived from water-soluble organic compounds such as flavonoids and terpenoids [65]. The absorption peak at 1760 cm^−1^ was assigned to the carbonyl (C=O) stretching groups of *U. lactuca* proteins [35]. The presence of these functional groups in FTIR analysis further indicates the existence of important bioactive compounds in the *U. lactuca* extract, including polyphenols, flavonoids, terpenoids, polysaccharides, and proteins. Numerous studies have identified these phytochemicals as being responsible for the bioreduction and stability of NPs [7,66]. The FTIR spectra of photocatalytically biosynthesized LU-AgNPs exhibited three peaks at 3300 cm^−1^, 2100 cm^−1^, and 1628 cm^−1^. Compared to crude *U. lactuca* extract, there was a considerable decrease in the absorbance intensities and a shift in the absorbance peaks from 1760 cm^−1^ to 1628 cm^−1^, 2280 cm^−1^ to 2100 cm^−1^, and 3420 cm^−1^ to 3300 cm^−1^. The frequency variation in FTIR peaks between the extract and LU-AgNPs corresponds to the interaction or bonding of a functional group with the surface atoms of NPs [16]. The peak shifting and decrease in band intensities observed in the spectral profile of photocatalytically biosynthesized LU-AgNPs further confirmed the interactions between the phytochemical functional groups and LU-AgNPs [67]. The decrease in band intensity from 3420 cm^−1^ to 3300 cm^−1^ confirmed the involvement of OH^−^ and proteins in the bioreduction and stabilization of LU-AgNPs. The presence of a spectral peak at 2100 cm^−1^ suggests the involvement of secondary metabolites in the bioreduction and stabilization of LU-AgNPs. The key difference between the spectra of *U. lactuca* extract before and after Ag^+^ reduction is the change in the absorbance band from 1760 cm^−1^ to 1628 cm^−1^, which might be due to the stabilization effect of proteins of LU-AgNPs [68]. *U. lactuca* has a protein content of 27.94 ± 0.014 g/100 g of its dry weight, which supports the aforementioned statement. Biomolecules present in algal extracts play critical roles in capping and stabilizing NPs. Phenols (OH group) and proteins (carbonyl group) exhibit a strong affinity for silver and other metals, forming a layer that surrounds the NPs and results in capping [59,69]. This finding is substantiated by numerous additional studies, which also document the pivotal role played by hydroxyl and carboxylic acid groups as the two highly significant functional groups engaged in NP production [35,70,71]. Consistent with the outcomes of the present study, Fourier Transform Infrared (FTIR) analysis conducted on LU-AgNPs, synthesized using an aqueous extract of *U. lactuca*, revealed the presence of a multitude of functional groups. Among these groups are phenols, carbonyl compounds, carboxylic acids, amines, and various other significant functional groups. These findings strongly suggest that hydroxyl groups (O-H) play a vital role in the photoreduction of Ag^+^ induced by sunlight during the process of LU-AgNP biosynthesis. Additionally, other functional groups, including N-H and C=O, were detected on the surface of the NPs. These groups serve as stabilizing agents, effectively preventing the aggregation and agglomeration of the NPs and thereby ensuring their long-term colloidal stability.

#### 3.7.5. X-ray Diffraction (XRD) Analysis

**Figure 8:**
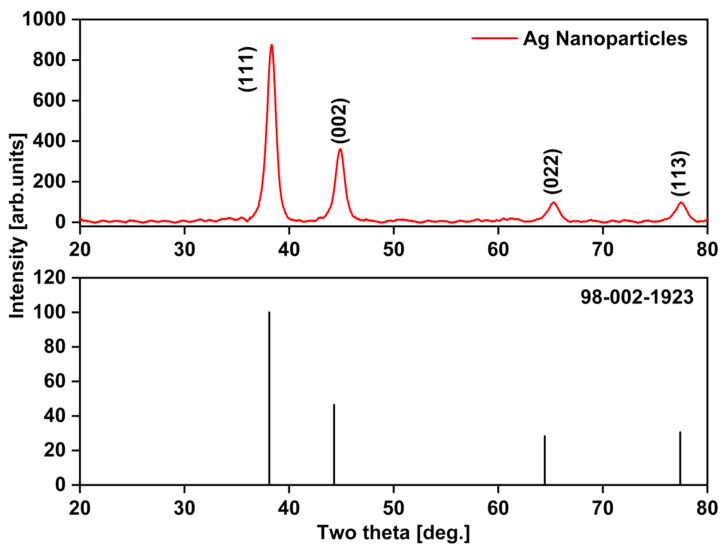
X-ray diffraction (XRD) spectra analysis of LU-AgNPs and Bragg’s reflection of the face-centered cubic (fcc) elemental silver (JCPDS No. 98-002-1923).

X-ray diffraction (XRD) was utilized to determine the crystallinity of the LU-AgNPs. Figure 8 illustrates the powder XRD pattern of LU-AgNPs, displaying four different diffraction peaks in a 2θ range from 0 to 80. The observed diffraction peaks at 38.34°, 44.91°, 65.29°, and 77.47° can be indexed to (111), (200), (220), and (311) crystallographic planes, respectively, in accordance with Bragg’s reflection of the face-centered cubic (fcc) structure of Ag, as documented in the database of the Joint Committee on Power Diffraction Standards (JCPDS; code number: 98-002-1923) [72,73]. LU-AgNPs exhibit intense peaks, with the peak at 2θ = 38.34° being the most prominent, implying a preferred orientation along the (200) lattice plane. Similar findings were reported for the green synthesis of AgNPs using carrageenan powder [72], callus extracts of *Allophylus serratus* [74], and seaweed *Sargassum polycystum* [75].

#### 3.7.6. Dynamic Light Scattering (DLS) of Photoinduced Biosynthesized AgNPs

Table 2 depicts the dynamic light scattering (DLS) size distribution histogram of photocatalytically biosynthesized LU-AgNPs, showcasing a size range spanning from 25 to 250 nm, with an average particle size (PS) of 43 nm. It is worth noting that the average particle size slightly exceeded the diameter values derived from TEM micrographs. The particle size distribution curve reveals the polydispersed nature of LU-AgNPs, with the majority of particles measuring less than 100 nm. This observation corroborates the findings of the TEM analysis (Figure 4), highlighting the presence of LU-AgNPs of varying sizes in the solution. Our obtained LU-AgNPs exhibited a polydispersity index of 1.092, thereby confirming the broad particle size distribution. Assessing the polydispersity index is vital for elucidating the nanoparticle size distribution. A polydispersity index (PI) value close to one signifies a heterogeneous particle size distribution, while a value of zero signifies a monodisperse population [76]. The average size values of our photocatalytically biosynthesized LU-AgNPs varied between the TEM and DLS analyses due to the influence of Brownian motion and the presence of capping molecules attached to the surface, which move with the LU-AgNPs in the aqueous colloidal suspension [67]. This difference is possibly due to particle agglomeration caused by the heterogeneous aggregated dispersion behavior of the LU-AgNPs [49] and the hydrated state of the sample [77]. Overall, our observations align with previous studies that have reported similar particle sizes using DLS analysis [78,79,80,81].

#### 3.7.7. Zeta Potential Analysis of Photoinduced Biosynthesized AgNPs

Zeta potential is a measure of surface charges and stability of AgNPs, which is a crucial parameter for various applications. A higher negative or positive zeta potential indicates greater stability and better colloidal properties, attributable to electrostatic repulsion and improved dispersibility. Our photocatalytically biosynthesized LU-AgNPs showed a zeta potential of −59.0 mV, indicating negatively charged surfaces in the biosynthesized LU-AgNPs (Table 3). A high negative potential value indicates the presence of negatively charged molecules in the extracts, resulting in NPs with electrostatic stability [82] and creating a strong repulsive force between them. This repulsive force prevents particle aggregation and promotes the wide dispersion of LU-AgNPs in the medium [77]. The negative zeta potential value also indicates the presence of some bio-organic capping molecules that prevent LU-AgNP agglomeration [37]. The FTIR analysis reveals the involvement of hydroxyl (O-H) and amine groups (N-H) in the reduction of Ag^+^ to Ag^0^. It also confirms that the negative zeta potential of LU-AgNPs is likely due to the presence of hydroxyl and amine group-containing phytoconstituents, which are responsible for the stability of LU-AgNPs. The ability of bio-organic constituents to function both as capping and reducing agents is especially important when the NPs are intended for biomedical applications.

#### 3.7.8. Mechanism/Biofunctional Groups Involved in Light-Induced Biocatalytic AgNP Synthesis

In order to understand the mechanism behind the light-induced biosynthesis of LU-AgNP synthesis, it is essential to identify the biomolecules of *U. lactuca* responsible for the reduction of Ag^+^ to Ag^0^. The analysis of FTIR spectra of *U. lactuca* extract further revealed the presence of polysaccharides, polyphenols, and proteins, which contain functional groups known to donate electrons for the reduction process (Figure 7). Among the various functional groups, –OH groups are known to reduce metal ions to their corresponding metal NPs. The phytochemical analysis showed a high concentration of polysaccharides, suggesting that carbohydrates may have sufficient–OH groups for Ag^+^ reduction. However, carbohydrate molecules are composed of long chains of monosaccharide residues, and each long chain of carbohydrates contains only one reducing carboxyl group, which cannot provide sufficient reducing power to produce AgNPs [83]. Additionally, the polysaccharides present in the cell walls of macroalgae serve a protective role against environmental stress and are not involved in any light absorption mechanism [84]. Therefore, the abundance of phenolic compounds in *U. lactuca* could provide a better explanation for the reduction of Ag^+^ to Ag^0^ (Table 1). Phenolic compounds, such as quinoids and saponins, act as secondary metabolites in most algae, mitigating photoinhibition and photooxidative damage. These compounds contain multiple–OH groups that can readily release electrons for the photoreduction of Ag^+^ to Ag^0^ when exposed to sunlight. Chlorophylls and carotenoids, which are other light absorption compounds present in algae, may not be present in the aqueous extract and, therefore, may not contribute to the formation of LU-AgNPs. It is likely that sunlight facilitates the transfer of electrons from the −OH groups of organic phenolic compounds to reducible Ag^+^ ions, thereby facilitating the synthesis of AgNPs [4]. This prediction is supported by our FTIR analysis, which indicates the involvement of –OH and –NH_2_ groups in the synthesis and stabilization of LU-AgNPs (Figure 7).

### 3.8. Catalytic Redox Potential of Photocatalytically Biosynthesized LU-AgNPs—Dye Degradation Activity

Dye pollutants pose a threat to water and soil systems, putting surrounding ecosystems at risk [85]. Various reductants have been utilized to convert these hazardous dyes into harmless intermediates [86]. However, this process is often inefficient and time-consuming. Recent developments have introduced the use of nano catalysts for the removal of pollutant dyes, benefiting from their larger surface area for an enhanced reaction [87]. In our study, we investigated the photocatalytic degradation of pollutant dyes, such as AO, CR, CBB G-250, MO, and RB, using photoinduced biomolecule-assisted AgNPs as photocatalysts under sunlight. Additionally, we conducted a kinetic analysis to understand degradation and decolorization pattern over time. The catalytic degradation rate of the metallic nanoparticles followed a pseudo-first-order reaction [88]. Hence, we employed the equation ln(A_0_/A*_t_*) = *kt*, where *k* is the reaction rate constant, A_0_ and A*_t_* are the initial absorbance and absorbance at time *t*, to express the rate [8].

#### 3.8.1. Degradation of RB

**Figure 9:**
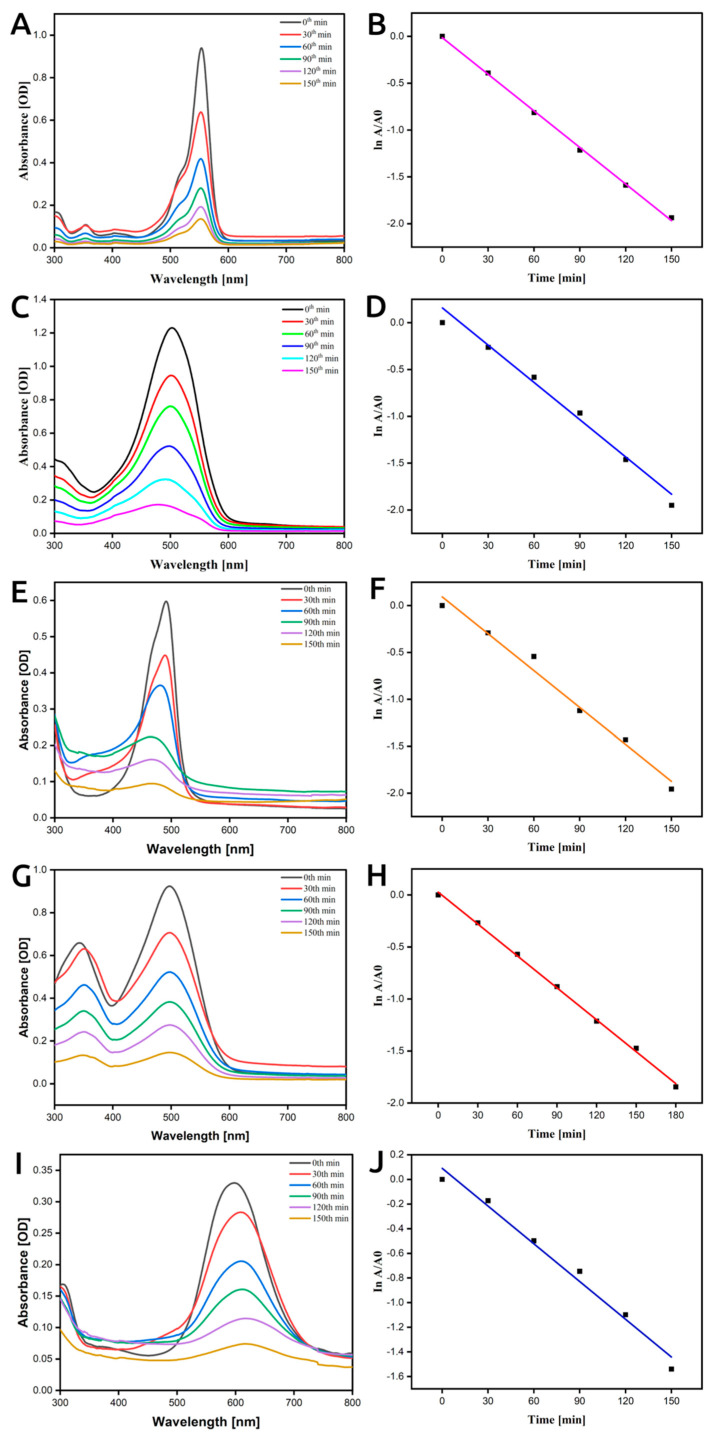
Catalytic redox potential of photocatalytically biosynthesized LU-AgNPs-dye degradation studies. (**A**) UV-vis spectra for photocatalytic degradation of RB using LU-AgNPs, (**B**) ln(A/A_0_) vs. time for the reduction of RB using LU-AgNPs, (**C**) UV-vis spectra for photocatalytic degradation of MO using LU-AgNPs, (**D**) ln(A/A_0_) vs. time for the reduction of MO using LU-AgNPs, (**E**) UV-vis spectra for photocatalytic degradation of AO using LU-AgNPs, (**F**) ln(A/A_0_) vs. time for the reduction of AO using LU-AgNPs, (**G**) UV-vis spectra for photocatalytic degradation of CR using LU-AgNPs, (**H**) ln(A/A_0_) vs. time for the reduction of CR using LU-AgNPs, (**I**) UV-vis spectra for photocatalytic degradation of CBB G-250 using LU-AgNPs, and (**J**) ln(A/A_0_) vs. time for the reduction of CBB G-250 using LU-AgNPs. AO: acridine orange, CBB G-250: Coomassie brilliant blue G-250, CR: Congo red, MO: methylene orange, RB: rhodamine B.

RB is a fluorescent dye widely used in industries such as paper, textile, and food, but it poses risks as a neurotoxic and carcinogenic metal-chelating reagent [89]. Conventional wastewater treatment methods often fail to effectively remove these contaminating pigments [63]. In our study, we conducted the photocatalytic degradation of RB in an aqueous solution under sunlight irradiation to evaluate the photocatalytic activity of our biosynthesized LU-AgNPs. We utilized a UV-vis spectrophotometer with a wavelength range from 300 to 800 nm, as shown in Figure 9A. The intense characteristic peak around 554 nm, corresponding to the pink appearance of the RB solution, decreased over time. Figure 9B illustrates the plot of Ln(A/A_0_) vs. time for catalytic degradation of RB by AgNPs. The calculated degradation rate constant for RB was 1.3024264 × 10^−2^ m^−1^. Our results align with a previous study where AgNPs synthesized from *Matricaria chamomilla* L. leaf extracts demonstrated promising catalytic activity, achieving approximately 93.37% degradation of RB after 130 min of UV irradiation [4]. However, in our experiment, the degradation of RB (85%) was relatively slower as it was conducted under sunlight with a lower concentration of LU-AgNPs (10 mg/100 mL), while the aforementioned study employed UV illumination, providing stronger irradiation with a higher AgNP concentration of 50 mg/mL.

#### 3.8.2. Degradation of MO

MO is a commonly used azo dye and a wastewater pollutant that poses potential hazards to the environment [90]. Therefore, efficient treatment methods for MO removal are highly desirable. In our degradation study, the absorption band for MO existed at 460 nm, as shown in Figure 9C. The absorption band of MO at 460 nm gradually faded with different time intervals after the addition of LU-AgNPs as a catalyst, as shown in Figure 9C. Plotting the relative absorption intensity with wavelength at regular time intervals revealed that the degradation of MO occurred within 3 h in the presence of LU-AgNPs as a catalyst. The plot of Ln(A/A_0_) vs. time for the catalytic degradation of MO by LU-AgNPs is depicted in Figure 9D, and the calculated degradation rate constant of MO was 1.3268309 × 10^−2^ m^−1^. Other studies reported that MO degradation occurred within 24 h in the presence of AgNPs as a catalyst, but the calculated degradation rate constant was 1.86 × 10^−3^ min^−1^, which was much slower compared to our LU-AgNPs [91].

#### 3.8.3. Degradation of AO

To evaluate the catalytic activity of our photocatalytically biosynthesized LU-AgNPs, we conducted the reduction of AO (a water contaminant) using green-synthesized LU-AgNPs at various time intervals while monitoring the process using UV-vis spectroscopy (Figure 9E). The absorption peak for AO dye in water was observed at 520 nm. In the presence of LU-AgNPs as a catalyst, the absorption spectrum showed a decrease in peaks for AO with time. The reduction of AO dye was evident from the gradual decrease in the absorbance values, approaching the baseline. Eventually, the absorbance disappeared with increasing reaction time, indicating efficient and complete degradation of AO dye by LU-AgNPs. The increase in dye removal depends on the presence of more active sites on the surface of the NPs [92]. A plot of ln(A/A_0_) vs. time for catalytic degradation of AO by LU-AgNPs is depicted in Figure 9F, and the calculated degradation rate constant value of AO was 1.3121891 × 10^−2^ min^−1^. In a previous study, the degradation of AO using AgNPs produced from an aqueous extract of *Erigeron bonariensis* by employing varying volumes of 1 mM of Ag colloids (extract + AgNO_3_, 1 mL, 2 mL, and 3 mL), which were exposed under sunlight for 180 min [41]. The results showed that 3 mL of Ag colloid completely degraded the AO within 50 min, which was a concentration of nearly 1.7 mg/mL. In contrast, our LU-AgNPs, at a lower concentration of 0.1 mg/mL, were sufficient to achieve complete degradation of AO within 130 min (Figure 9F).

#### 3.8.4. Degradation of CR

The sodium salt of the dye 3,3′-([1,1′-biphenyl]-4,4′-diyl)bis (4-aminonaphthalene-1-sulfonic acid) has a negative impact on aquatic habitats due to its carcinogenic properties [93]. Here, we investigated the catalytic degradation of CR-containing wastewater using AgNPs as catalysts. Figure 9G illustrates the absorption band of CR appearing at 519 nm. The degradation of CR was monitored by a UV-vis spectrophotometer, and a plot of Ln(A/A_0_) versus time for catalytic degradation of CR is depicted in Figure 9H. The results indicated that the rate of CR degradation was observed through a decrease in peak intensity within 3 h of incubation time. The calculated degradation rate constant value for CR was 1.02 × 10^−3^ min^−1^. In a study conducted by David and Moldovan [88], they used fruit extract of *Viburnum opulus* L.-derived AgNPs to degrade CR and MO in the presence of NaBH_4_, obtaining rate constants of 0.0795 and 0.1178 min^−1^ for the degradation of CR and MO, respectively. Their reported degradation rate was higher than ours due to the usage of NaBH_4_ for the stability and reusability of AgNPs, while in our present study, no additives were used.

#### 3.8.5. Degradation of CBB G-250

The distinctive absorption peak of CBB G-250 at 595 nm was utilized to monitor the catalytic degradation process. Figure 9I displays the absorption spectra of CBB G-250 in an aqueous solution and in the presence of Ag nanoparticles at different time intervals. As the exposure time increased, the maximum absorption peak at 595 nm gradually decreased, indicating the photocatalytic degradation of CBB G-250. Figure 9J shows the plot of Ln(A/A_0_) vs. time for catalytic degradation of CBB G-250 by AgNPs, and the calculated degradation rate constant value of CBB G-250 was 1.0210749 × 10^−2^ min^−1^. Arunachalam et al. [94] synthesized AgNPs from *Coccinia grandis* leaf extract to degrade CBB G-250 and reported that the degradation process gradually increased with an increase in exposure time. Our current research demonstrates significantly different degradation potentials of LU-AgNPs for CBB G-250 based on kinetic studies.

#### 3.8.6. Mechanism of LU-AgNP-Mediated Redox Reaction for Dye Degradation

The mechanism of dye degradation could be attributed to the surface plasmon resonance (SPR) effect of the LU-AgNPs since none of the controls have shown any dye degradation when just exposed to sunlight. When the electrons in the biocatalyst are exposed to sunlight, they are excited and move from the valence band to the conduction band. These high-energy electrons form electron-hole pairs, which lead to the production of free radicals like hydroxyl and anions of oxygen reacting with H_2_O and O_2_, respectively. Subsequently, these excited free radicals break down the organic dye into simple organic molecules, resulting in dye degradation [95,96]. Jaast and Grewal [92] reported that light illumination accelerates the generation of electron-hole pairs, thereby increasing the reduction and oxidation processes of dyes. According to Alshehri and Malik [4], the radicals like O_2_^−•^, OH^−^, and HO_2_^•^ generated through light exposure can degrade RB and promote its transformation to less toxic fragments such as NH_4_^+^, CO_2_, NO_3_^−^ and water molecules. The catalytic degradation of the AO occurs likely through an N-de-methylation pathway involving the attack of ^•^OH radicals, which is a powerful oxidant [9]. In addition, AgNP dye degradation activity is also affected by their spherical structure and size. Smaller particles have a larger surface area and more active sites, resulting in stronger binding strength. Previous studies have demonstrated that the decolorization of dyes under sunlight is faster in the presence of a metal catalyst compared to other irradiation techniques [17,37,92,94]. AgNPs are highly efficient and stable photocatalysts under ambient temperature and visible light illumination [16]. Therefore, LU-AgNPs can be utilized as highly efficient and stable photocatalysts to degrade organic dyes. The photocatalytic activity of LU-AgNPs directly depends on their crystallographic structure, morphology, and size [16].

In addition to their dye degradation potential, photoinduced phycogenic LU-AgNPs have great potential for biomedical applications. Biogenic AgNPs have been extensively studied and found effective for various applications, including antibacterial, antifungal, antiviral, anti-cancer, anti-angiogenic, and anti-inflammatory applications, as well as dental material filling, vaccine adjuvants, antidiabetic treatments, bioimaging, and drug delivery [97]. Biogenic AgNPs are mainly utilized for their antimicrobial properties, especially in combating multi-drug-resistant microorganisms, as traditional antimicrobial chemicals may lose their efficacy. The specific redox properties of NPs play a crucial role in their impact on biological entities [98]. For instance, photocatalytically biosynthesized LU-AgNPs can adhere to the surface of microbial cell walls, penetrate into the cells, and damage intracellular organelles such as mitochondria, ribosomes, DNA, and proteins by inducing oxidative stress through the generation of reactive oxygen intermediates (ROIs), ultimately leading to the death of microbes [99]. Similarly, AgNPs synthesized using *Durio zibethinus* seed extract have demonstrated remarkable antibacterial properties against both Gram-positive and Gram-negative bacteria [37]. AgNPs have also exhibited good antifungal activity against various plant pathogenic fungi, including *Colletotrichum coccodes*, *Monilinia* sp., and *Candida* spp., in a dose-dependent manner [100]. Interestingly, AgNPs with a diameter smaller than 10 nm have shown remarkable antiviral activity against many viruses, including hepatitis B virus (HBV), human parainfluenza virus (HPIV), herpes simplex virus (HSV), influenza A (H1N1), and human immunodeficiency virus (HIV), again due to its larger surface area, which facilitates stronger adhesion to the virus. Furthermore, when combined with antimicrobial agents, biogenic AgNPs show drug-sensitizing effects [101]. Likewise, plant-based AgNPs apparently show ROI-mediated toxicity through metabolic changes in cancer cells [102]. The generation of ROIs, such as peroxide (H_2_O_2_) and superoxide (O_2_^−^) radicals, can disrupt the transmembrane potential of mitochondria, leading to the uncoupling of the respiration mechanism, oxidative stress, and ultimately, cell death [103]. Moreover, photocatalytically biosynthesized AgNPs may exhibit significant antidiabetic and anti-inflammatory activities. Biosynthesized AgNPs have been demonstrated to have antidiabetic effects both in vitro and in vivo by lowering blood glucose levels and inhibiting the action of three enzymes: dipeptidyl peptidase [104], α-amylase, and α-glucosidase [105]. Additionally, AgNPs can induce an anti-inflammatory response during wound healing by modulating the expression of various inflammatory response genes, including tumor necrosis factor (TNF)-α, interferons, interleukin 1 (IL-1), cyclooxygenase (COX)-2, and matrix metalloproteinases-3 [106]. Chemical-mediated AgNPs show promise for use in the biomedical sector [107] primarily due to their biocompatibility and antibacterial activity. Many of these AgNPs have received approval for clinical trials by the United States Food and Drug Administration (FDA). The photoinduced phycogenic LU-AgNPs produced in the present study are considered eco-friendly and possess suitable characteristics for various future biomedical applications.

## 4. Conclusions

The photoinduced biosynthesis of AgNPs utilizing the aqueous extract of the marine green alga *U. lactuca* has proven to be an elegant, expeditious, cost-effective, environmentally benign, and facile green approach. Parameters such as light intensity, wavelength, reaction time, and pH play pivotal roles in influencing the morphology, size, and synthesis rate of AgNPs. The resulting LU-AgNPs were meticulously characterized through UV-vis, TEM, SAED, EDX, AFM, FTIR, and DLS analyses, revealing predominantly spherical shapes, crystalline structure, polydispersity, and exceptional stability, with an average diameter of 15 nm. The bioreduction and stability of AgNP formation were ascribed to the presence of algal polyphenols and proteins. Our research also unraveled a pathway for identifying the biomolecule responsible for light absorption, offering a promising avenue for the development of rapid commercial synthesis techniques for AgNPs. Our LU-AgNPs exhibited remarkable photocatalytic properties, showcasing their potential for degrading a diverse range of water-soluble dye pollutants. Furthermore, kinetic studies substantiated the excellent degradation potential of LU-AgNPs for each dye, achieving rapid degradation. Additionally, modifications such as NP immobilization could prove invaluable in utilizing these materials in polluted water environments. Consequently, the photocatalytic biosynthesis of LU-AgNPs utilizing *U. lactuca* extract holds tremendous promise for large-scale synthesis in the bioremediation of wastewater systems, owing to its high reproducibility and efficiency. Moreover, the photocatalytically biosynthesized LU-AgNPs could serve as a valuable resource for biomedical redox reaction applications [108,109,110].

## Figures and Tables

**Table 1:** Proximate phytochemical composition of *U. lactuca* (g/100 g of dry weight).

Carbohydrate	Protein	Lipid	Phenol	Flavonoid
37.27 ± 0.012	27.94 ± 0.014	10.29 ± 0.010	0.07 ± 0.015	0.05 ± 0.012

**Table 2:** Dynamic light scattering (DLS) unveils a fascinating size distribution of photocatalytically biosynthesized LU-AgNPs, spanning from 25 to 250 nm. These extraordinary nanoparticles exhibit an average particle size of 43 nm, exemplifying their remarkable properties.

Peak No.	P.S. Area Ratio	Mean	S.D.	Mode
1	1.00	76.1 nm	34.1 nm	60.7 nm
2	-	-	-	-
3	-	-	-	-
Total	1.00	76.1 nm	34.1 nm	60.7 nm

Z-Average: 43.0 nm. Polydispersity Index (PI): 1.092. S.D.: Standard deviation. P.S.: Particle size.

**Table 3:** The zeta potential distribution of photocatalytically biosynthesized LU-AgNPs shows that the LU-AgNPs carry highly negative surface charges.

Peak No.	Zeta Potential	Electrophoretic Mobility
1	−59.0 mV	−0.000456 cm^2^/Vs
2	-	-
3	-	-

## Data Availability

The data is contained within the article.
